# An ERP study on facial emotion processing in young people with subjective memory complaints

**DOI:** 10.1038/s41598-021-90861-9

**Published:** 2021-05-31

**Authors:** Vanesa Perez, Ruth Garrido-Chaves, Mario Perez-Alarcón, Tiago O. Paiva, Matias M. Pulopulos, Vanesa Hidalgo, Alicia Salvador

**Affiliations:** 1 Laboratory of Social Cognitive Neuroscience, IDOCAL, Department of Psychobiology, University of Valencia, Valencia, Spain; 2 Laboratory of Neuropsychophysiology, Faculty of Psychology and Education Sciences, University of Porto, Porto, Portugal; 3 Department of Medical Imaging, University of Porto, Porto, Portugal; 4 Department of Psychology and Sociology, Area of Psychobiology, University of Zaragoza, IIS Aragón, Teruel, Spain

**Keywords:** Neuroscience, Physiology, Psychology

## Abstract

Subjective memory complaints (SMCs) are commonly related to aging, but they are also presented by young adults. Their neurophysiological mechanisms are not thoroughly understood, although some aspects related to affective state have been mentioned. Here, we investigated whether facial emotion processing is different in young people with (n = 41) and without (n = 39) SMCs who were exposed to positive, negative, and neutral faces, by recording the event-related potential (ERP) activity. From the ERP activity, the N170 (an index of face processing) and the LPP (an index of motivated attention) components were extracted. Regarding the N170, results showed less amplitude for positive and neutral faces in the participants with SMCs than in those without SMCs. Moreover, women with SMCs displayed longer latencies for neutral faces than women without SMCs. No significant differences were found between the groups in the LPP component. Together, our findings suggest deficits in an early stage of facial emotion processing in young people with SMCs, and they emphasize the importance of further examining affective dimensions.

## Introduction

Subjective memory complaints (SMCs) have been defined as subjective awareness of memory loss in the absence of any organic or identifiable condition in neuropsychological examinations^1^. A previous review pointed out that the prevalence of SMCs among older people ranges from 25 to 50%, depending on the assessment method and the population´s characteristics^2^. In Spain, the prevalence of SMCs was 32.4% in a study conducted with older people in Madrid^3^, which is similar to the prevalence observed in a large community-based study in Australia^4^. However, although memory complaints are frequently reported by older people, the available evidence indicates that SMCs are also reported by young adults^5,6^. A study carried out in the UK reported that the prevalence of SMCs increased with age, and 5.5% to 6.3% of people between 16 and 24 years old reported SMCs^7^. As in older people, research in young people has shown that SMCs are not associated with objective memory performance^8,9^. However, SMCs have been related to attention and executive difficulties^10^, perceived stress^8,11^ and anxiety symptoms^12–14^ in young adults, and to depression in a mixed-age sample^15^. In this regard, it is important to investigate the mechanisms that may contribute to an increased perception of stress and even the development of stress-related disorders, such as anxiety and depression, in young adults with SMCs.

Facial emotion processing is a complex process that involves many cerebral structures, including the amygdala and occipitotemporal cortex and, particularly, the orbitofrontal cortex^16^. The latter region is known to play an important role in attention to emotional stimuli^17^. Taking into account that correct facial emotion processing is necessary for successful human interactions^18^, its impairment could at least partly contribute to the development of stress-related disorders reported in people with SMCs^19–21^. In addition, facial emotion processing is an important source of knowledge about the emotions of others and social information (for a review see:^22^) that is commonly affected in older people with SMCs^23,24^. The ability to efficiently recognize emotional facial expressions correlates with problem-solving capacity and facilitates social interactions and adequate adaptation to a new environment^25,26^. Moreover, some studies have shown that, in disorders related to cognitive impairment, deficits in the facial emotion processing ability influence social behavior (e.g., the ability to perceive and recognize the affective state of others)^27,28^. Along these lines, previous studies have shown a relationship between attentional bias toward negative stimuli and anxiety^20,29^, long-term stress^30^, and depression^21^ in young people. Therefore, investigating deficits in facial emotion processing in young people with SMCs may be interesting because it would help to understand the development of stress-related disorders and provide potential evidence of difficulties in attending to emotional stimuli in this population.

Facial emotion processing can be analyzed by event-related potentials (ERPs)^22^, which are considered reliable biomarkers of cognitive operations^23^ that allow the assessment of neural reactivity to affective events with a high temporal resolution^31^. Electrophysiological data reveal that ERPs are sensitive to the emotional content of facial expressions in early stages of emotional processing^22^. More specifically, the main components related to faces and facial emotion processing are the N170^22^ and the Late Positive Potential (LPP)^21^. The N170 component is an early negative component that is detected at 120–200 ms and peaks at approximately 170 ms post-stimulus, which has been proposed as a correlate of the interpretation of a visual stimulus such as a face, although it can be induced by other objects consciously interpreted as face-like (e.g., pareidolia)^32,33^. Located primarily in the occipitotemporal brain region, the N170 usually shows a greater response over the right hemisphere than over the left hemisphere^34^, and it can be modulated by emotion processing^35^. Importantly, Lazarou et al.^23^, in their study on negative faces (anger and fear), demonstrated that older people with SMCs show larger N170 amplitudes to negative faces than healthy controls. The LPP component is a slow positive potential that occurs at approximately 400–600 ms post stimulus onset, with a maximum peak at the midline central and parietal electrodes, and it shows higher amplitudes for emotional images than for neutral images^36,37^, which represents sustained attention^20^. This component reflects brain electrical activity during both automatic and controlled attentional processing for emotional information, and it indicates more elaborate emotion-related processing, such as high-level recognition processing^38,39^. To the best of our knowledge, no previous studies have investigated the LPP component on a facial emotion processing task in people with SMCs.

With all this in mind, the present study aimed to investigate whether facial emotion processing is different in young people with and without SMCs who were exposed to positive, negative, and neutral faces. To do so, we employed the two main components of processing usually studied in this context, and we also included behavioral data (reaction time, RT, and accuracy). Given that higher amplitudes and shorter latencies are observed when attentional resources are abundant, and that processing in the N170 and LPP is sensitive to attentional resources^40^, we expected longer latencies and smaller amplitudes in both the N170 and LPP components in participants with SMCs, compared to those without SMCs. Finally, sex-related differences in facial emotion processing have been found, with faster recognition times, better accuracy, shorter latencies, and greater N170 and LPP amplitude in women than in men^41–43^. Therefore, we included both women and men in order to explore possible sex-related differences in young people with and without SMCs.

## Material and methods

### Participants

Eighty healthy young students participated in the study (41 men, 39 women; mean age = 22.1 years) (Table 1). Participants were recruited at the University of Valencia campus (Spain). Undergraduates who met the criteria were contacted by telephone and asked to attend a session that took place in the Laboratory of Social Cognitive Neuroscience.

**Table 1:** Demographic data (mean and SD) for each group and sex.

	SMCs	noSMCs	Men	Women
Age (years)	21.17 (3.27)	23.22 (4.0)	22.87 (3.89)	21.46 (3.53)
BMI (kg/m^2^)	21.93 (3.26)	23.16 (3.89)	23.10 (3.96)	21.96 (3.17)
SES	5.7 (1.22)	6.0 (1.14)	6.0 (1.20)	5.71 (1.16)

*BMI* body mass index, *SES* socioeconomic status, *SMCs* subjective memory complaints.

The exclusion criteria were: history of alcohol or drug abuse; smoking more than 10 cigarettes a day; having had surgery under general anesthesia in the past year; presence of severe vision or hearing problems or an illness that involves an alteration of the nervous system; and a neurological or psychiatric disorder. In addition, participants were excluded if they took drugs that might affect cognitive or emotional function, psychotropic substances, beta-blockers, or benzodiazepines, or if they had experienced a stressful event in the past six months. All the participants were right-handed according to the Edinburgh Handedness Inventory^44^.

Participants were distributed into two groups: SMCs (N = 41; 20 men and 21 women) and no SMCs (noSMCs) (N = 39; 21 men and 18 women), according to the scores obtained on the Spanish adaptation^6^ of the modified version of the Memory Failures of Everyday (MFE-30) questionnaire^45^. This questionnaire consists of 30 items about situations and activities of daily life, rated on a 5-point Likert scale ranging from 0 (never or almost never) to 4 (always or almost always). We employed these scores to distribute participants into two groups according to the scores obtained on this questionnaire. The participants who scored equal to or below 21 were included in the noSMCs group, whereas the participants who scored above 21 were included in the SMCs group (descriptive data for each condition and sex group are summarized in Table 1).

The study was carried out according to the Declaration of Helsinki, and the Ethics Committee of the University of Valencia approved the protocol. All the participants received verbal and written information about the study and signed an informed consent.

### Procedure

Participants arrived at the laboratory, and the experimenter verified that they had followed the instructions given before the experiment: sleep as long as usual, refrain from heavy physical activity the day before the session, and not consume alcohol or any stimulants since the night before the session.

The experimental session took 2 h and was carried out in the morning (between 10 and 14:00 h) or in the afternoon (between 15:00 and 19:00 h). Half of the participants attended the morning shift, and the other half attended the afternoon shift. There were no differences in the number of participants in each group in each shift (χ^2^ = 2.8 *p* = 0.423).

The session started with a period of habituation to the laboratory lasting 15 min. Then, the participants were prepared for the EEG register (10 min) and performed two blocks of resting EEG collection (i.e. closed eyes and opened eyes) for three minutes each block. Next, the face stimulus task was presented, which lasted 12 min. After the task, weight and height were measured, and then the participants had 40 min to answer the MFE-30 and a General Questionnaire where demographic data were collected. As part of a larger research project not related to the research question of the current study, the participants completed other cognitive tasks and questionnaires (data not included here).

### Face stimulus task

Images of human facial expressions with positive, negative, and neutral valences were used as stimuli. Each valence contained 68 images. All the images were adapted from a standard set of pictures to generate emotional stimuli^46^. Images of men and women and the valences of the images were presented randomly to the participants and in equal proportion. All the images were presented in grayscale on a black background and displayed in the center of a 24-inch screen.

**Figure 1:**
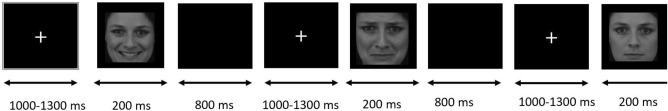
Timeline of events during the session. Note: *ms* milliseconds. The images are an example of faces of each emotion extracted from the Karolinska Emotional Directed Faces database. Image ID: AF01DIS, AF01ANS, AF01NES. Copyright: https://kdef.se/home/using%20and%20publishing%20kdef%20and%20akdef.html.

The stimuli were presented in the following sequence: (1) a fixation mark ( +) appeared for 1000 to 1300 ms; (2) the face was presented for 200 ms; and (3) a blank screen was displayed for 800 ms (Fig. 1). The images were presented using the E-prime program (v2.0). Participants were instructed to press the 1 key if the facial expression was positive, 2 if it was negative, and 3 if it was neutral. The participants were seated 70 cm from the screen in a dimly lit and sound attenuated room. The task started with practice trials containing 12 images. Each participant received feedback after each of these 12 trials, indicating whether he/she had done it correctly or incorrectly.

### ERP recording and data analyses

The EEG data were collected using an elastic cap from a 29-channel system, according to the international 10–20 system (Fp1, Fpz, Fp2, F7, F3, Fz, F4, F8, A1, T3, C3, Cz, T4, C4, A2, T5, P3, Pz, P4, T6, O1, OZ and O2), using a Brain Vision Amplifier System (Brains product, Germany). The electrode AFz was used as the system ground, and electrodes were referenced to Fcz. Both vertical and horizontal electro-oculograms were captured by additional electrodes (VEOG-, VEOG + , HEOG-, HEOG +) placed around the eyes. The electrode-to-skin impedances were lowered using electrolyte gel (SUPER-VISC High Viscosity Electrolyte-Gel, EasyCap, GmbH), and they were kept below 5 kΩ before starting the recording. The BrainVision Analyzer (BrainProducts, Germany) was used to analyze the EEG data. Data were re-referenced to a common average signal of 23 electrodes^47^. The EEG and EOG were amplified and then passed through (0.1–30 Hz) band-pass filtering using an IIR filter (24 db/octave roll-off). One-second epochs were extracted in a range from − 200 to 800 ms. Epochs were then corrected to the mean voltage of the baseline − 200. Trials with EOG artifacts, including blinking, eye movement, and skin potentials, were corrected offline with the algorithm from Gratton and Coles^48^, and trials with wrong answers were removed from averaging. Based on the overall mean chart, the early ERP component (i.e. N170) generated by the stimuli showed clear peaks. A time window of 130–200 ms was used to measure the ERP peak and peak latency in data collected at electrode sites T6 (right temporal lobe) and T5 (left temporal lobe) for N170. The LPP component was calculated at the Pz electrode, with a mean value of the amplitude within a 400–700 ms time-window.

### Statistical analyses

For behavioral performance, RT and accuracy were analyzed using ANOVAs for mixed-designs, with Emotional Valence (positive, negative, and neutral) as a within-subject factor and Sex (men and women) and Group (SMCs and noSMCs) as between-subject factors.

To study the amplitudes and latencies of the N170 and LPP components, we carried out ANOVAs for mixed-designs, with Emotional Valence (positive, negative, neutral) as a within-subject factor and Sex and Group as between-subject factors. In the analyses with N170, Hemisphere (Right and Left) was also included as a within-subject factor.

In cases of violation of sphericity, Greenhouse–Geisser correction was applied. Post-hoc planned comparisons were performed using Bonferroni adjustments for the p values. The level of significance was taken as *p* = 0.05. There were no outliers (± 3SD) in this study. We used SPSS 24.0 to perform the statistical analysis.

## Results

### Behavioral performance

Table 2 shows RT and accuracy. For RT, a significant effect of Emotional Valence, F (1.963, 149.189) = 204.095, *p* = 0.001, ηp^2^ = 0.729, was found. RT were shorter for positive faces than for negative and neutral faces (both *p* < 0.001), and shorter for negative faces than for neutral (*p* = 0.005) faces. Neither the Sex (*p* = 0.627) and Group (*p* = 0.349) factors nor their interactions were significant (all *p* > 0.994).

**Table 2:** Means and standard deviations for the behavioral performance for each group and sex.

	SMCs	noSMCs		Men	Women
**Reaction time (ms)**					
Positive	684.8 (75.8)	664.9 (86.9)		669.7 (79.3)	680.8 (84.4)
Negative	807.7 (85.2)	789.9 (107.4)		798.3 (88.0)	799.7 (102.7)
Neutral	833.6 (99.3)	813.8 (116.8)		814.6 (107.3)	833.7 (109.2)
**Response accuracy (%)**					
Positive	94.6 (2.9)	95.8 (2.0)		94. 9 (2.9)	95.6 (2.1)
Negative	77.5 (11.6)	79.7 (5.9)		79.5 (6.4)	77.6 (11.5)
Neutral	80.5 (14.1)	87.8 (6.4)		87.6 (5.7)	80.3 (14.7)

*ms* milliseconds, *SMCs* subjective memory complaints.

For accuracy, a significant effect of Emotional Valence, F (1.973, 149.964) = 51.899, *p* < 0.001, ηp^2^ = 0.406, was found. Thus, accuracy was higher for positive faces than for negative and neutral faces (both *p* < 0.001), and it was higher for neutral faces than for negative faces (*p* = 0.003). Neither the main effects of Sex (*p* = 0.190) and Group (*p* = 0.089) nor the rest of the interactions reached statistical significance (*ps* > 0.542).

### ERP data analysis

#### N170 component

**Figure 2:**
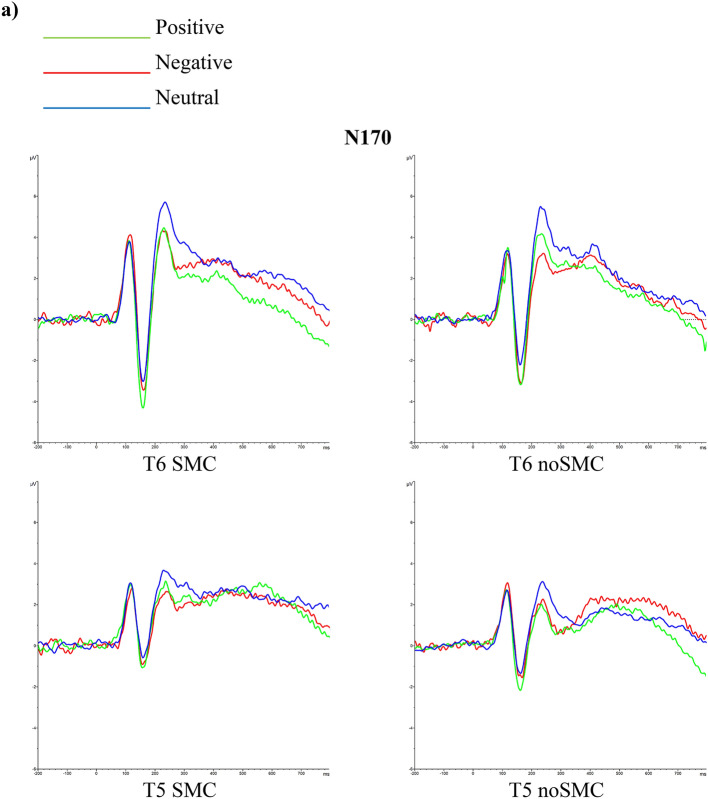

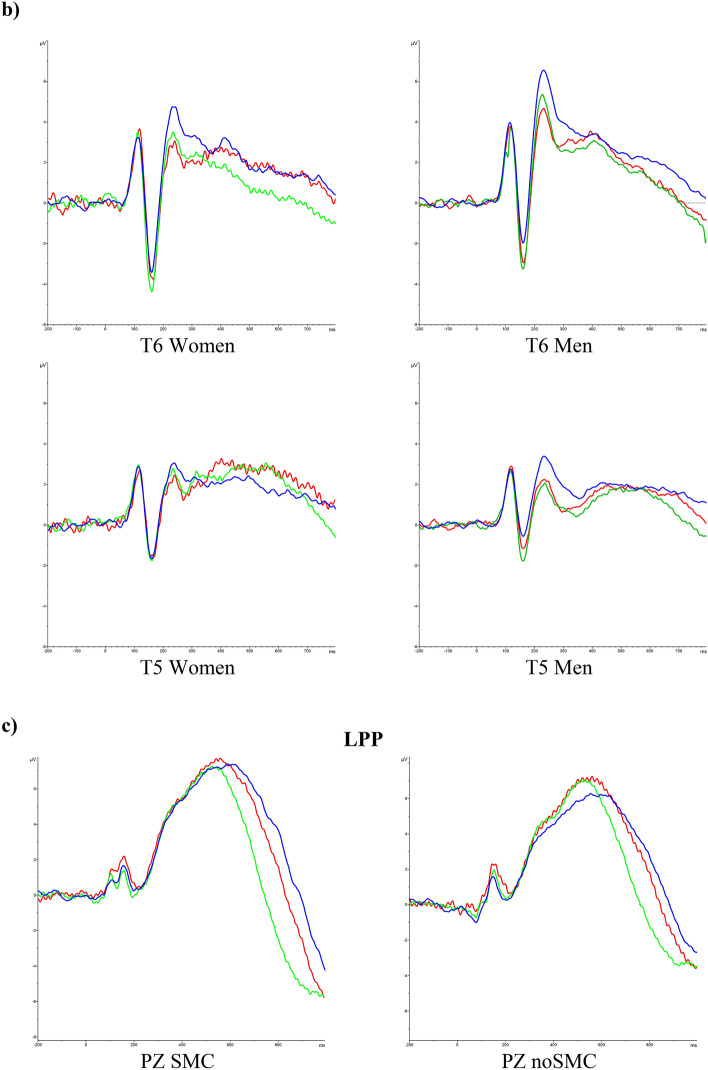

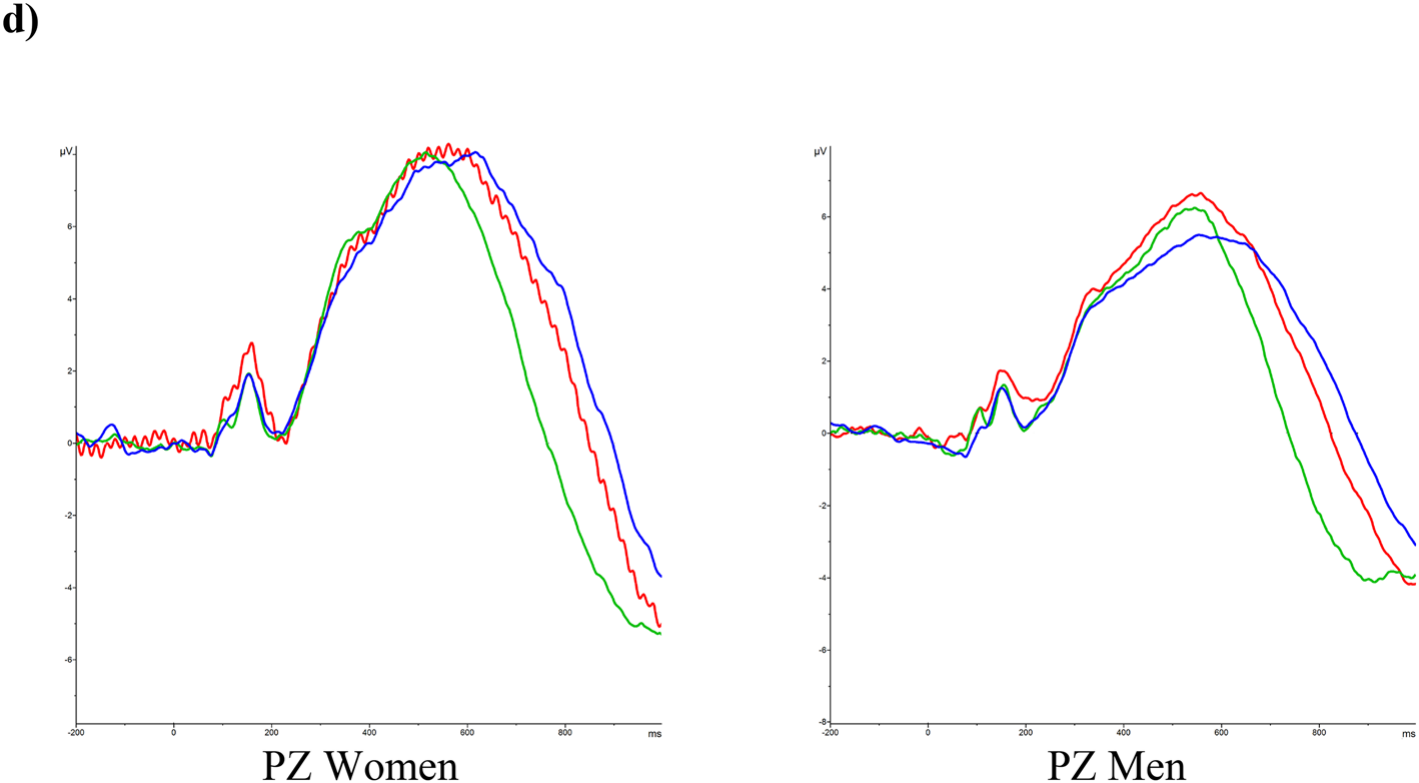
Latencies and amplitudes of N170 and LPP induced by groups and sex. (**a**) Grand average N170 for positive (green), negative (red), and neutral (blue) faces recorded in the right and left hemisphere in young people with and without subjective memory complaints. (**b**). Grand average N170 for positive, negative, and neutral faces recorded in the right and left hemisphere in young women and men. (**c**). Grand average LPP for positive, negative, and neutral faces in young people with and without subjective memory complaints. (**d**). Grand average LPP for positive, negative, and neutral faces recorded in young women and men.

For N170 latencies, the analyses revealed that the Emotional Valence, F (1.955, 136.858) = 1.553 *p* = 0.216, ηp^2^ = 0.216, Hemisphere, F (1,70) = 0.615 *p* = 0.436, ηp^2^ = 0.009, Sex, F (1, 70) = 0.353 *p* = 0.554, ηp^2^ = 0.005, and Group, F (1, 70) = 1.338 *p* = 0.251, ηp^2^ = 0.019, factors were not significant. The Emotional Valence x Group interaction, F (1.955, 136.858) = 3.256 *p* = 0.043, ηp^2^ = 0.044, was statistically significant; however, post hoc comparisons did not show significant differences (all *ps* > 0.926) (Fig. 2a). The Emotional Valence x Hemisphere x Group x Sex interaction was also significant, F (1.878, 131.4556) = 3.226 *p* = 0.046, ηp^2^ = 0.044. Post hoc analyses revealed that women SMCs showed longer latencies in the right hemisphere for neutral faces than women noSMCs (*p* = 0.005). None of the other post hoc analyses revealed significant effects (all *ps* > 0.082). The other interactions were not significant (*ps* > 0.764).

For N170 amplitudes, the effect of Emotional Valence was significant, F (2, 140) = 21.274 *p* = 0.001, ηp^2^ = 0.233, with higher amplitudes for positive faces than for negative *(p* = 0.049) and neutral (*p* = 0.001) faces, and higher amplitudes for negative faces than for neutral (*p* = 0.001) faces. The Group factor was also significant, F (1, 70) = 4.563 *p* = 0.036, ηp^2^ = 0.061, indicating that SMC participants showed less amplitude than noSMCs. In addition, a significant effect of the Emotional Valence x Group interaction, F (2, 140) = 5.331 *p* = 0.006, ηp^2^ = 0.071, was found. Post hoc comparisons revealed that the SMC participants showed lower amplitudes than noSMCs for positive (*p* = 0.007) and neutral (*p* = 0.050) faces, but not for negative faces (*p* = 0.148) (Fig. 2a).

We also found a significant effect of the Hemisphere, F (1, 70) = 17.550 *p* < 0.001, ηp^2^ = 0.200, and Sex, F (1, 70) = 4.200 *p* = 0.044, ηp^2^ = 0.057, factors (Fig. 2b). Thus, we observed higher amplitude in the right hemisphere than in the left hemisphere, and men showed less amplitude than women. Other interactions were not statistically significant (*ps* > 0.711).

#### LPP component

For LPP latencies, results showed a significant effect of Emotional Valence, F (2, 136) = 9.475 *p* < 0.001, ηp^2^ = 0.122. LPP latency was shorter for positive faces compared to neutral (*p* = 0.001) faces, and shorter for negative faces compared to neutral (*p* = 0.034) faces. Other effects or interactions were not significant (*ps* > 0.614).

For LPP amplitudes, the Emotional Valence was significant, F (1.912, 131.924) = 5.431 *p* = 0.006, ηp^2^ = 0.073. Post hoc comparison revealed that amplitudes were significantly higher for negative faces than for positive (*p* = 0.003) faces, but no other significant differences were found (all *p* > 0.232) (Fig. 2c).

In addition, the Sex factor was significant, F (1, 69) = 5.261 *p* = 0.025, ηp^2^ = 0.071, with men showing smaller LPP amplitudes than women. Other effects and interactions were not significant (*ps* > 0.827) (Fig. 2d).

## Discussion

The aim of the present study was to investigate whether facial emotion processing (i.e. positive, negative, and neutral faces) is different in young women and men with SMCs compared to other groups without SMCs. At the behavioral level, only positive valences showed clearly significant effects on both RT and accuracy, with no significant differences due to SMCs or sex. Regarding ERPs, we found that participants with SMCs showed lower amplitudes than noSMC participants in the N170 component, specifically for positive and neutral faces. In addition, women SMCs showed longer latencies in N170 for neutral faces compared to women noSMCs. For the LPP component, no differences depending on SMCs were found for latency and amplitude. Notably, the participants showed higher amplitudes for negative faces. Finally, we observed that women showed higher amplitudes than men for both the N170 and LPP components, although no differences in latencies were found.

Regarding behavioral data, neither RT nor accuracy was significantly different between the SMCs and noSMCs groups, indicating similar performance for both groups. This lack of differences between groups in the behavioral data is consistent with a previous study carried out in controls and older people with SMCs^24^. Regardless of the group and sex, participants showed less latency and more accuracy for positive expressions, and they were slower for neutral faces and less accurate for negative faces. Complementary to this, Calvo and Lundqvist^49^ demonstrated that positive expressions have a salient and unique facial feature, the smile, which allows quick and accurate identification. By contrast, negative or neutral expressions contain more overlapping features, which would generate confusion, making the decision process slower^49^.

Although there is debate about the meaning of the N170 component, it may be considered a neural indicator of facial structure encoding^32,50^, where the structural representation of the face is associated with the necessary semantic information to form an internal representation of a human face^50^, combining both bottom-up and top-down processes. In addition to structure encoding, the N170 is also involved in facial emotion processing, especially for the early processing of the emotional valence^51^. In this vein, the smaller amplitude elicited in N170 by the SMCs group compared to the noSMCs group might reflect emotional processing difficulties in this stage of processing in this population. Moreover, on closer examination, the N170 amplitude was lower for positive and neutral faces in SMCs than in noSMCs, whereas negative faces elicited similar amplitudes in both groups. From a biological point of view, a possible interpretation of this pattern of processing of negative faces could have an adaptive function, given that negative face recognition is more relevant because these expressions are signs of potential harm^52^. To the best of our knowledge, no previous studies have investigated the N170 component with positive, negative, and neutral faces together in relation to SMCs. One study carried out in older people with a diagnosis of SMCs, Alzheimer disease, mild cognitive impairment, and healthy older participants only focused on negative stimuli to capture any cognitive changes^23^. These authors found larger N170 amplitudes in response to faces showing fear in SMCs than in healthy participants, suggesting that an increase in N170 amplitudes reflects the difficulty in computing spatial relations among face features and understanding the different negative emotions of facial expressions. However, these results must be interpreted with caution because some studies have indicated that low amplitudes (in regions involved in face processing, particularly in frontal and temporal areas) would be related to a decline in emotion processing^53^. Furthermore, because this study only takes negative facial stimuli into consideration, its results may not be used for comparisons with positive or neutral stimuli. We also found that there is right hemisphere dominance for N170 amplitude in both groups. This result supports evidence showing that neurons in the right superior temporal gyrus respond to the processing of facial expressions of emotion (^32^, see review:^54^).

Regarding the LPP, we found no differences between the SMCs and noSMCs groups in latency or in amplitude. However, regardless of the group, participants presented shorter latencies for positive faces than for negative and neutral faces, whereas the amplitude was greater for negative faces than for positive and neutral faces. This result is consistent with our behavioral data showing slower and less accurate responses for negative faces. In agreement with this, prior research suggests that positive faces are more easily processed in this late stage^52^. In addition, more attentional resources and greater cortical activation are allocated to negative faces than to positive and neutral faces in this stage of processing^40^. This probably occurs because negative faces have greater biological relevance, and so the attention is directed toward these significant stimuli. In the present study, young people with SMCs showed a deficit in attentional resources only for early processing of facial positive and neutral emotions (N170), whereas both groups processed negative stimuli similarly. These findings are consistent with the hypothesis that the processing of potentially threatening stimuli may occur without attentional involvement^40^. Together, our results suggest that young people with SMCs would not present difficulties in late processing, which involves a greater evaluation of the information related to the affective valence of a face^40^; that is, sustained attention would be preserved.

The relationship between SMCs and facial emotion processing is complex and poorly understood. Some studies indicate that impaired emotional face processing affects quality of life and interactions in everyday social life^25,27^. Consequently, impaired facial emotion processing can have a negative impact on social behavioral competence^18^, and it could play an important role in the development of stress-related psychopathology in young people with SMCs.

Finally, we found that men showed smaller amplitudes for both the N170 and LPP components than women. Several studies have investigated the relationship between sex and facial emotion processing, but the results are mixed. Some did not observe sex differences^55^, other studies indicated that advantages women have over men are only for female faces and not for male faces (for a review see:^56^), whereas others observed sex differences^57^. Our finding could suggest that, in general, women show a more sensitive attentional processing of emotional faces than men. In addition, this processing begins in the first stage and is maintained in the late evaluative process. This result is in line with studies that have shown that women are more responsive to face stimuli than men, which could suggest greater empathy or greater attention to facial features and more interest in social information^57^.

Despite the relevance and novelty of our results, some limitations should be considered. First, further studies may benefit from investigating the effect of the arousal of emotional expressions when exploring the LPP component because valence mainly modulates the early stage of emotional processing, whereas arousal mainly modulates the late stage (for review see:^31^). Second, we used static and grayscale facial pictures as stimuli, and they subtract important lively information that people use to recognize facial expressions in natural contexts. In contrast, colors and three-dimensional stimuli provide a more real effect than our stimuli^58^. Finally, the power analysis in G*Power showed that the sample size is large enough to observe small to medium effect sizes (f = 145; power = 0.8, alpha = 0.05) for the main aim of the study, that is, the Emotional Valence x Group interaction. In fact, the results of this interaction indicated a medium to large effect size (ηp^2^ = 0.071, f = 0.276). However, given that it is not possible to calculate the sample size needed to detect a triple interaction (i.e., Emotional Valence x Sex x Group), we can assume that we only have enough statistical power to detect large effect sizes. Therefore, the results of the exploratory analyses investigating sex-related differences should be interpretated with caution. Finally, despite some strengths of this study, such as the thorough selection of the participants and the sample size, the number of statistical analyses carried out calls for caution in interpreting the results obtained and emphasizes the need to replicate them^59^.

In conclusion, our study showed subtle differences in N170 amplitudes for positive and neutral faces, which suggests that young adults with SMCs have difficulties in the early stage of emotional processing. Importantly, these difficulties were not observed in behavioral performance or in the late stages of emotional processing, which suggests that sustained attention to emotional faces is preserved in this age group. Further studies investigating the course of facial emotion processing would help us to understand some difficulties that characterize this population and that could be the cause of greater vulnerability to developing subjective deficits and other stress-related disorders.
